# Profiling of Antimicrobial Resistance Genes and Integron from *Escherichia coli* Isolates Using Whole Genome Sequencing

**DOI:** 10.3390/genes14061212

**Published:** 2023-06-01

**Authors:** Harshrajsinh B. Joddha, Rafiyuddin A. Mathakiya, Kuldip V. Joshi, Ravindra B. Khant, Akash V. Golaviya, Ankit T. Hinsu, Mansi R. Desai, Subhash J. Jakhesara, Prakash G. Koringa

**Affiliations:** 1 Department of Veterinary Microbiology, College of Veterinary Science and A H, Kamdhenu University, Anand 388001, Gujarat, India; hbjoddha333@gmail.com (H.B.J.); drramathakiya@gmail.com (R.A.M.); kuldipjoshi455@gmail.com (K.V.J.);; 2 Department of Animal Biotechnology, College of Veterinary Science and A H, Kamdhenu University, Anand 388001, Gujarat, India; ankit4035hinsu@gmail.com (A.T.H.); drsubhash81@gmail.com (S.J.J.)

**Keywords:** *Escherichia coli*, whole genome sequencing, ARGs, integron

## Abstract

This study is designed to investigate *Escherichia coli* for the antibiotic resistance genes (ARGs) and integrons from healthy as well as diarrhoeic/diseased animals/birds’ faecal samples. A total of eight samples were selected for the study; from each animal, two samples were taken, one from healthy animals/birds and one from diarrhoeic/diseased animals/birds. Antibiotic sensitivity testing (AST) and whole genome sequencing (WGS) was performed for selected isolates. The *E. coli* isolates showed resistance to moxifloxacin, followed by erythromycin, ciprofloxacin, pefloxacin, tetracycline, levofloxacin, ampicillin, amoxicillin, and sulfadiazine (4/8, 50.00% each). The *E. coli* isolates were 100% sensitive to amikacin, followed by chloramphenicol, cefixime, cefoperazone, and cephalothin. A total of 47 ARGs from 12 different antibiotic classes were detected among the eight isolates by WGS. The different classes of antibiotics included aminoglycoside, sulphonamide, tetracycline, trimethoprim, quinolone, fosfomycin, phenicol, macrolide, colistin, fosmidomycin, and multidrug efflux. The class 1 integrons were detected in 6/8 (75.00%) isolates with 14 different gene cassettes.

## 1. Introduction

*E. coli* in both animals and humans can frequently cause gastro-intestinal infection [1]. Since it may cause serious infections in humans and animals and, on the other hand, makes up a sizeable portion of the autochthonous microbiota of the various hosts, *E. coli* holds a special place in the field of microbiology. The greatest concern is the possibility of virulent and resistant *E. coli* spreading between humans and animals via numerous routes. *E. coli* is a significant source of resistance genes that may be to blame for both human and veterinary medical treatment failures [2].

The evolution and selection of bacteria that are resistant to antibiotics is significantly influenced by the use of antibiotics in animals for both medicinal and non-therapeutic purposes. As a result of chromosomal changes or horizontal gene transfer (HGT) of ARGs between related or unrelated bacteria, antimicrobial resistance (AMR) is currently the most pressing issue in bacteria, especially in members of the Enterobacterales family. This allows pathogenic bacteria to resist antimicrobial treatments and persist in hostile environments such as the gastrointestinal tract [3]. In HGT, transformation, transduction, and conjugation are the way by which genetic exchange takes place between two organisms, and in the presence or absence of antibiotics, certain genomic processes occur [4]. AMR, particularly, multidrug resistance (MDR), has become increasingly widespread in clinical isolates, including *E. coli* isolates from animals [5]. Sadly, the situation with bacteria developing resistance is worsening every day, and we have actually reached the stage where we can say that antimicrobial resistance is a major issue on a global scale [6].

Mobile genetic elements (MGEs) play a major role in HGT between the organism, host, and environment [7]. The horizontal transfer of AMR genes is often facilitated by MGEs such as plasmids, transposons, integrons [8], and integrative and conjugative elements (ICE) [9]. Integrons are a platform for acquiring open reading frames (ORFs) and converting them into functional forms via site-specific recombination [10]; thus, integrons can capture genes’ cassettes and express them as proteins (Figure S1) [11]. The bacterial save our souls (SOS) and stringent responses activate the integron integrase, which is initiated in response to both DNA damage and nutrition deprivation [12]. By frequently integrating in plasmids or transposons, integrons can spread horizontally in bacterial populations (Figure S2) [13]. Integrases are classified into many classes based on their aminoacidic sequence. Classes 1, 2, and 3 (*intI1*, *intI2*, *intI3*) were the first to be identified as being associated with MGEs, while class 4 (*intI4*) was associated with chromosomal integration [14].

As many antibiotic resistance genes (ARGs) are responsible for AMR in pathogens, high throughput and robust screening technology such as next generation sequencing (NGS) is required. The NGS technologies greatly benefit monitoring that rely on the characterization of genetic data [15]. These technologies make DNA and RNA sequencing significantly faster and less expensive than Sanger sequencing, which was previously employed.

The aim of this study is to determine the AMR genes present in the *E. coli* and to compare the numbers of genes present in the healthy and diarrhoeic/diseased animals/birds, because when an animal has diarrhoea, there is a presence of both pathogenic and non-pathogenic bacteria in the gut. Furthermore, integrons are also included in the study, because as a MGE, they play an important role in the transfer of genes from pathogenic bacteria to non-pathogenic bacteria.

## 2. Materials and Methods

### 2.1. E. coli Isolates

The *E. coli* isolates used in the present study were isolated in the previous study at the Department of Microbiology, Veterinary College, Kamdhenu University, Anand. There was a total of eight groups, which included diarrhoeic/diseased samples and samples from healthy cattle, buffalo, dogs, and poultry. From each group, one representative sample was selected for the present study.

### 2.2. Revival of E. coli Isolates

A total of eight *E. coli* isolates from different animals and poultry were selected. The isolates were preserved in glycerol at −40 °C and inoculated on MacConkey agar and incubated for 24 h at 37 °C (Figure S3). Isolated lactose-fermenting, pink-coloured colonies were inoculated on Eosin-Methylene Blue (EMB) agar for confirmation using greenish metallic sheen (Figure S4) and incubated for 24 h at 37 °C. Further, isolated pure culture colonies were inoculated on Brain Heart Infusion (BHI) agar (Figure S5) and stored for further study. The sample names are listed in Table 1.

### 2.3. Antibiotic Susceptibility Testing (AST) of E. coli Isolates

The selected eight isolates were subjected to AST using 20 different antibiotics from various antibiotic classes viz. cefixime (CFM, 5 mcg); cefoperazone (CPZ, 75 mcg); cefotaxime (CTX, 30 mcg); ceftriaxone (CTR, 30 mcg); cephalothin (CEP, 30 mcg); chloramphenicol (C, 30 mcg); amikacin (AK, 30 mcg); amoxicillin (AMX, 30 mcg); ampicillin (AMP, 25 mcg); ciprofloxacin (CIP, 1 mcg); co-trimoxazole (COT, 25 mcg); erythromycin (E, 15 mcg); gentamicin (GEN, 10 mcg); levofloxacin (LE, 5 mcg); moxifloxacin (MO, 5 mcg); pefloxacin (PF, 5 mcg); spectinomycin (SPT, 100 mcg); streptomycin (S, 10 mcg); sulfadiazine (SZ, 300 mcg); and tetracycline (TE, 30 mcg).

The pure cultured *E. coli* colonies from BHI agar were inoculated in BHI broth and incubated at 37 °C for 4–6 h. The turbidity of broth culture was adjusted to 0.5 McFarland standard (~1.5 × 10^8^ cfu/mL). After adjusting the turbidity, the sterile swab was dipped into broth and used to prepare a lawn culture on Mueller–Hinton Agar (MHA). The antibiotic discs were then placed on the agar plate and incubated for 24 h at 37 °C. On the next day, diameter of the zone of inhibition was measured and compared with zone size interpretative chart provided by the manufacturer and as of Clinical Laboratory Standard Institute (CLSI) (Figure S6).

### 2.4. Whole Genome Sequencing

#### 2.4.1. Extraction of DNA Using QIAamp DNA Mini Kit

DNA was extracted from the isolated pure cultured colonies from fresh culture plate using the QIAamp DNA mini kit according to the manufacturer’s instructions.

#### 2.4.2. Library Preparation

The quality of the extracted DNA was checked using a Qubit 3.0 Fluorometer (Thermo Fisher Scientific, Waltham, MA, USA). The library preparation was performed using a Nextera XT DNA Library Preparation kit. After library preparation, quantity and quality were checked using a Qubit 3.0 Fluorometer and Bioanalyzer 2100 (Agilent Technologies, Santa Clara, CA, USA) using high sensitivity DNA 1000 chip, respectively (Figure S8). Sequencing was performed using Illumina’s reagent kit v3 to produce a 2 × 300 bp pair-end on an Illumina MiSeq benchtop sequencer (Illumina, San Diego, CA, USA).

### 2.5. Whole Genome Data Assembly

The quality of raw data was checked using FastQC (version 0.11.9), while the low-quality sequence data were removed using PRINSEQ-lite (version 0.20.4), where the minimum limit for Q score was 20. Cutadapt (version 3.3) was used for the adaptor trimming. De novo assembly was performed on the CLC genomic workbench 22, where the minimum contig length was set to 1000. The quality of the contigs data was checked using QUEST (version 5.0.2).

#### 2.5.1. Detection of AMR Genes

For the detection of AMR genes from the contigs data, NCBI’s AMRFinderPlus [16] was used. AMRFinderPlus can detect acquired antibiotic resistance, stress response, virulence genes, and genetic mutations that are known to confer antibiotic resistance. AMRFinderPlus relies on the NCBI’s curated Reference Gene Database and curated collection of Hidden Markov Models (HMMs). The Reference Gene Catalog, database version 2022-05-26.1, was used.

#### 2.5.2. Detection of Integron

The Integron Finder (version 2.0.1) tool [17] was used to detect the integrons in the sequenced samples.

## 3. Results

### 3.1. Antibiotic Susceptibility Testing (AST) Result

AST was performed to check the phenotypic resistance of the bacteria. A total of 20 antibiotics were used for AST; among them, the isolated *E. coli* isolates were found to be the most resistant to moxifloxacin (8/8, 100%), followed by erythromycin (7/8, 87.50%), ciprofloxacin, pefloxacin, tetracycline (6/8, 75.00% each), levofloxacin, and ampicillin (5/8, 62.50% each). The *E. coli* isolates were highly sensitive to amikacin (8/8, 100%), followed by chloramphenicol (6/8, 75.00%), cefixime, cefoperazone, and cephalothin (5/8, 62.50% each) (Tables S1 and S2). The isolate which was found to be resistant to 19 out of 20 antibiotics was CD13, followed by BD11 and DD9 (*n* = 15 each), PD14 (*n* = 11), PH1 (*n* = 9), DH9 (*n* = 5), CH13 (*n* = 3), and BH8 (*n* = 2) (Figure S7).

### 3.2. Quantification and Quality Checking of Prepared Library

The Qubit 3.0 fluorometer was used for the quantification of the prepared libraries, and the Bioanalyzer 2100 (Agilent Technologies, Santa Clara, CA, USA) with the Agilent DNA 1000 kit was used to check the library size distribution. The library concentration for the samples ranges from 10.5 ng/µL (CH13) to 15.1 ng/µL (DH9). The average library size for the samples ranges from 1500 bp (DH9) to 1719 bp (PH1) (Table 2).

### 3.3. Whole Genome Sequencing Analysis

The data were generated using Illumina MiSeq, and the coverage for each sample ranged from 100× to 173× (Table 3). The WGS assembly was performed using the CLC genomic workbench 22. In the final assembly, the contig numbers ranged from 41 to 262, with a minimum length of 1000 bp.

### 3.4. Detection of Antimicrobial Resistance Genes (ARGs) Using AMRFinderPlus

In the present study, AMRFinderPlus was used to detect ARGs in the *E. coli* isolates. A total of 47 ARGs from 12 different antibiotic classes (Table 4) were detected among the eight isolates using the AMRFinderPlus tool. The different classes of antibiotics were aminoglycoside (*aph(3″)-Ib or strA*, *aph(6)-Id or strB*, *aadA1*, *ant(2″)-Ia*, *aadA5*, *aph(4)-Ia*, *aadA2*, *aac(3)-Iva*, *aac(3)-VIa*, *aac(3)-IId*, *aac(6′)-Ib-cr5*, and *aac(3)-IIe*); β-lactam (*bla*EC, *bla*_OXA-1_, *bla*_TEM_, *bla*_CTX-M-15_, and *bla*_TEM-1_); sulphonamide (*sul1*, *sul2*, and *sul3*); tetracycline (*tet(A)* and *tet(B)*); trimethoprim (*dfrA5*, *dfrA17*, and *dfrA36*); quinolone (*qnrS1*, *gyrA_S83L*, *parC_S80I*, *gyrA_D87N*, *parE_I529L*, *parE_I355T*, *parC_E84G*, and *parC_E84V*); and fosfomycin (Table 5).

The highest number of ARGs were found in the DD9 isolate (*n* = 21), followed by the PH1 isolate (*n* = 19), CD13 isolate (*n* = 18), PD14 isolate (*n* = 16), BD11 isolate (*n* = 15), DH9 isolate (*n* = 12), and the CH13 isolate and BH8 isolate (*n* = 6 each). When we compare the healthy and diarrhoeic isolates for ARGs, the number of ARGs were higher in the diarrhoeic isolates than in the healthy isolates except in the poultry isolates, where the healthy isolates had more ARGs than the diseased isolates.

### 3.5. Detection of Integrons Using Integron Finder Tools

Integrons play an important role in the transfer of ARGs between different bacteria. In the present study, the class 1 integron was detected in six out of eight isolates (Table 6). There was a total 14 gene cassettes which were related to the class 1 integron viz. *dhfrA17*, *aadA5*, *ant(2”)-Ia*, *aac(3)-VI*, *aac(6)-Ib*, *aadA*, *aadA1*, *aadA2*, *emrE*, *catB*, *cmlA1*, *bla*_OXA-1_, *qacL*, and *qacEdelta1*. None of the isolates were found to be positive for the class 2 integron.

The highest number of gene cassettes (*n* = 5) were found in the DD9 isolate, followed by the PH1 (*n* = 4), BD11 and CD13 (*n* = 3), and PD14 (*n* = 2) isolates.

## 4. Discussion

### 4.1. Antimicrobial Susceptibility Testing Using Disc Diffusion Method

Antibiotics have the ability to inhibit the growth of bacteria or kill the bacteria. However, when these bacteria can survive in the presence of antibiotics, this is known as antibiotic resistance. Phenotypically, we can check the susceptibility of a bacteria to antibiotics. There are various methods for this, and one commonly used is the disc diffusion method, which was used in the present study.

Among the collected *E. coli* isolates, six isolates showed resistance to ciprofloxacin. Similar studies have shown resistance ranging from 60% to 92% [18,19,20,21]. In this study, 75% of the isolates showed resistance to tetracycline. One study reported 100% resistance to tetracycline in the *E. coli* isolated from broiler chicks and calves [21,22]. In the case of erythromycin, the observed resistance was 87.50%, and other studies have shown resistance ranging from 80% to 100% in the *E. coli* isolates from diseased poultry birds [18,23,24].

The present study is designed to measure the differences between the antibiotic resistance of the *E. coli* isolates from healthy and diarrhoeic/diseased animals/birds. The diarrhoeic isolates showed more resistance compared to the healthy isolates. The *E. coli* isolates from poultry showed a different scenario, where both (healthy and diseased) isolates showed no such difference in the antibiotic resistance. In the present study, the *E. coli* isolates that recovered from diarrhoeic cattle showed resistance to 19 antibiotics, which was higher than that of any isolate.

### 4.2. ARG Detection among Different E. coli Isolates

In the present study, a total of 47 AMR genes were identified, of which 34 genes were without chromosomal mutations and 13 were with chromosomal mutation, which makes 13 other genes (Table S3). All the isolates showed resistance to multiple classes of antibiotics, ranging from 3 to 10. Only two isolates had six AMR genes. Other isolates had AMR genes in the range from 12 to 21. Based on the findings of the present study, all the isolates were multidrug resistant.

From the aminoglycoside class, the *strA* and *strB* gene were detected in 37.50% of the isolates. Other similar studies have found *strA* and *strB* in *E. coli* isolates from different animals [7,25,26]. The *aac(6′)-Ib-cr5* gene was found only in *E. coli* isolates from diarrhoeic dogs, which confers resistance to aminoglycoside as well as fluoroquinolone, and similar studies have also detected *aac(6′)-Ib-cr5* in dog and calf samples [27,28].

The quinolone resistance genes detected in the isolates were *qnrS1* and *gyrA*, and *parC* and *parE* (with chromosomal mutation). Other studies have also detected *qnrS1* genes and genes with chromosomal mutations in *E. coli* isolated from cattle and poultry [29,30,31,32]. Bacteria having β-lactam-resistant genes produce β-lactamase enzymes that hydrolyse the β-lactam ring of antibiotics from the class β-lactam. In this study, *bla*_OXA-1_, *bla*_CTX-M-15_, and *bla*_TEM-1_ were detected. *bla*_CTX-M-15_ is an extended-spectrum β-lactamase. Similarly, β-lactam resistance genes have also been detected from *E. coli* isolates from different animals [26,32,33,34,35].

Resistance to sulphonamides occurs principally through the acquisition of the alternative dihydropteroate synthase (DHPS) gene *sul*, the product of which has a low affinity for sulphonamides [36]. In the present study, three *sul* genes were detected. Similarly, sulphonamide resistance genes were detected in the *E. coli* in previous studies [28,35,37]. In the present study, trimethoprim resistance genes (*dfrA*) were found. Similarly, previous studies have also found trimethoprim resistance genes in *E. coli* isolated from cattle and poultry [30,31,33].

Two tetracycline resistance genes (*tetA* and *tetB*) were detected among eight *E. coli* isolates, wherein the *E. coli* isolated from diseased poultry harboured the *tetB* gene, and all other isolates harboured the *tetA* gene. Similarly, previous studies detected tetracycline resistance genes in *E. coli* isolated from cattle and poultry [28,37,38].

### 4.3. Correlation between Phenotypic Resistance to Antibiotics and Presence of ARGs

In this study, streptomycin resistance genes (*strA* and *strB*) were found in three isolates, all of which showed phenotypic resistance to streptomycin. Thus, we can say that the genes present in the *E. coli* isolate are expressing themselves. Similarly, four isolates harboured gentamicin resistance genes (*ant(2”)-Ia*, *aac(3)-VIa*, *aac(3)-IIe*, *aac(3)-IId*, and *aac(3)-IVa*); among them, three isolates showed phenotypic resistance to gentamicin, and one isolate did not show phenotypic resistance.

In the case of β-lactam antibiotics, the *bla*_CTX-M-15_ gene confers resistance to ampicillin, amoxicillin, ceftriaxone, and cefotaxime. Three isolates harboured the *bla*_CTX-M-15_ gene and showed phenotypic resistance to the above-mentioned antibiotics. One isolate harboured the *bla*_OXA-1_ gene and showed phenotypic resistance to ceftriaxone. For tetracycline, six isolates were positive for the *tetA* and *tetB* gene, and all six isolates showed phenotypic resistance to tetracycline. Four isolates that were phenotypically resistant to co-trimoxazole had sulphonamide and trimethoprim resistance genes. Two isolates had resistance genes against sulphonamide and trimethoprim but did not show phenotypic resistance. Similar to chloramphenicol, two isolates possessed resistance genes and showed phenotypic resistance to chloramphenicol.

The isolates that possessed the ARGs and showed resistance indicated that the gene was expressed and showed resistance to a particular antibiotic for which it confers resistance. The isolates that did not show resistance to antibiotics but had ARG might be due to a lack of gene expression.

### 4.4. Intraspecies Discussion in Relation to Antimicrobial Susceptibility Testing

In the present study, the healthy buffalo and diarrhoeic buffalo samples show a high difference in the antibiotic resistance pattern. The healthy buffalo sample was found to be resistant to only 2 antibiotics, whereas the diarrhoeic buffalo samples were found to be resistant to 15 antibiotics.

The healthy cattle and diarrhoeic cattle samples also show a high difference in the antibiotic resistance pattern. The healthy cattle sample was resistant to 3 antibiotics, and the diarrhoeic cattle sample was found to be resistant to 19 antibiotics.

The healthy dog sample was found to be resistant to 5 antibiotics, whereas the diarrhoeic dog sample was found to be resistant to 15 antibiotics. The healthy poultry and the diseased poultry samples do not show much difference in the resistance against antibiotics. The healthy poultry samples show resistance to 9 antibiotics, and the diseased poultry samples show resistance to 11 antibiotics.

In the present study, the diarrhoeic/diseased samples were found to be more resistant to antibiotics when compared to the healthy samples, but in the poultry, there is not much difference shown between the healthy and diseased samples. Among all the diarrhoeic/diseased samples, the diarrhoeic cattle sample shows the highest resistance to a number of antibiotics (19 antibiotics). The healthy poultry samples show resistance against nine antibiotics, which is higher when compared to other healthy samples.

### 4.5. Intraspecies Discussion in Relation to ARGs Detected by AMRFinderPlus

In the present study, 6 ARGs were detected in the healthy buffalo isolate (BH8), whereas the diarrhoeic buffalo isolate (BD11) was found to be positive for 15 different ARGs. The healthy cattle (CH13) and diarrhoeic cattle (CD13) showed a difference in the detected ARGs. A total of 6 ARGs were found in the CH13 isolate, and a total of 18 ARGs were found in the CD13 isolate.

In the dog isolates, 12 ARGs were detected in the healthy dog (DH9) isolate, whereas the diarrhoeic dog isolate (DD9) was found to be positive for 21 ARGs. The healthy poultry (PH1) and diseased poultry (PD14) samples do not show much difference in the number of ARGs detected. In the healthy poultry isolate, 19 ARGs were detected, and in the diseased poultry isolate, 16 ARGs were detected.

Among the healthy isolates, the healthy poultry isolates carry the highest number of ARGs (19). The diarrhoeic dog sample carries the highest number of ARGs (21) among all the diarrhoeic/diseased samples. The diarrhoeic/diseased samples carried more ARGs when compared to the healthy samples, except the poultry samples, in which there is not much difference between the ARGs detected in the samples.

### 4.6. Detection of Integrons from Bacterial Genome

In the present study, the class 1 integron was found in 6/8 isolates (75.00%). The class 1 integrons were also detected from various animals (cattle, poultry, dogs, and cats) [7,22,35,39,40,41].

In the present study, among the isolates obtained from diarrhoeic/diseased animals or poultry, all the isolates were found to be positive for the class 1 integron (intI1), whereas among the isolates from the healthy animals/poultry, two isolates were positive for the class 1 integron (intI1). However, none of the isolates were positive for the class 2 integron (intI2).

## 5. Conclusions

This study was performed to determine the AMR genes in the *E. coli* isolates from healthy as well as diarrhoeic/diseased animals/birds. Overall, the results clearly show a difference in the number of AMR genes between the *E. coli* isolated from healthy and diarrhoeic/diseased animals/birds. The *E. coli* isolates from the healthy poultry harboured a greater number of AMR genes compared to the *E. coli* isolate of the diseased birds. In the present study, integrons are also detected, which plays an important role in the transfer of AMR genes between related or unrelated bacteria. Class 1 integrons were detected from all the isolates. In conclusion, whenever there is a diarrhoeic condition in the animal, at that time, normal flora comes into contact with more pathogenic bacteria that harbour many AMR genes, which can get transferred from the pathogenic bacteria to the normal flora of the animal with the help of the integron.

## Figures and Tables

**Table 1:** Details of *E. coli* isolates obtained from healthy and diarrhoeic faecal samples of buffalo, cattle, dogs, and poultry.

Sr. No.	Animal Species	Health Status	Sample Code
1	Buffalo	Healthy	BH8
2	Buffalo	Diarrhoeic	BD11
3	Cattle	Healthy	CH13
4	Cattle	Diarrhoeic	CD13
5	Dog	Healthy	DH9
6	Dog	Diarrhoeic	DD9
7	Poultry	Healthy	PH1
8	Poultry	Diseased	PD14

**Table 2:** The extracted DNA concentration and concentration after library preparation.

Sample ID	Extracted DNA Concentration (ng/µL)	After Library Preparation	
		DNA Concentration (ng/µL)	Average Size (bp)
BH8	34.8	14.4	1622
BD11	30.2	12.7	1590
CH13	32.4	10.5	1631
CD13	20.5	11.9	1550
DH9	17.2	15.1	1500
DD9	26.4	14	1603
PH1	19.8	11.6	1719
PD14	21.3	13.2	1508

**Table 3:** The summary statistics of the assembled draft genomes of *E. coli*.

Genomic Data	*E. coli* Isolates							
	BH8	BD11	CH13	CD13	DH9	DD9	PH1	PD14
	Healthy	Diarrhoeic	Healthy	Diarrhoeic	Healthy	Diarrhoeic	Healthy	Diseased
Raw reads	3,346,676	2,590,074	2,788,186	2,847,306	2,714,658	2,750,746	2,223,648	2,539,014
Read length	300	300	300	300	300	300	300	300
QC passed reads	2,774,938	2,156,170	2,290,014	2,353,304	2,289,542	2,346,802	1,754,196	2,205,784
GC percentage	50.80%	50.60%	50.80%	50.45%	50.87%	50.62%	50.55%	50.78%
Coverage	173×	118×	146×	125×	143×	132×	100×	130×
Average contig size	117,059	28,222	82,459	21,503	57,801	49,763	35,767	40,556
Largest contig	690,373	244,419	372,188	235,503	515,703	432,575	373,088	338,128
N50	307,169	97,172	171,274	77,091	128,318	155,303	103,877	105,260
L50	6	19	10	23	10	13	16	14
Total contig	41	194	57	262	83	107	147	125
Total bases	4,799,423	5,475,066	4,700,174	5,633,836	4,797,467	5,324,679	5,257,682	5,069,499

**Table 4:** Detection of antibiotic resistance genes and its comparison with antimicrobial susceptibility test.

c	Isolates Phenotypically Positive for Antibiotic Resistance (Disc Diffusion Method)		Isolates Genotypically Positive for Antibiotic Resistance Genes (WGS)		Sequence Accession Number
	Resistance to No. of Antibiotics	Name of Antibiotics	No. of Detected ARGs	Name of ARGs Detected in Isolate	
BH8	2	E, MO	6	*bla*EC, *glpT_E448K*, *emrD*, *mdtM*, *acrF*, *emrE*	JAOXMM000000000
BD11	15	AMX, AMP, CFM, CPZ, CTX, CTR, CEP, CIP, COT, E, MO, PF, S, SZ, TE	15	*aph(3”)-Ib*, *aph(6)-Id*, *aadA5*, *bla*EC, *bla*_CTX-M-15_, *sul1*, *sul2*, *tet(A)*, *dfrA17*, *qnrS1*, *mph(A)*, *pmrB_Y358N*, *glpT_E448K*, *acrF*, *mdtM*	JAOXMN000000000
CH13	3	AMP, E, MO,	6	*bla*EC, *glpT_E448K*, *pmrB_Y358N*, *mdtM*, *acrF*, *emrE*	JAOXMO000000000
CD13	19	AMX, AMP, CFM, CPZ, CTX, CTR, CEP, C, CIP, COT, E, GEN, LE, MO, PF, SPT, S, SZ, TE	18	*aph(3”)-Ib*, *aph(6)-Id*, *aadA1*, *ant(2”)-Ia*, *bla*EC, *bla*_TEM_, *bla*_CTX-M-15_, *sul1*, *sul2*, *tet(A)*, *dfrA5*, *dfrA36*, *qnrS1*, *gyrA_S83L*, *glpT_E448K*, *floR*, *acrF*, *mdtM*	JAOXMP000000000
DH9	5	CIP, LE, MO, PF, TE	12	*aac(3)-VIa*, *blaEC*, *sul1*, *tet(A)*, *parC_s80I*, *gyrA_D87N*, *gyrA_S83L*, *uhpT_E350Q*, *glpT_E448K*, *pmrB_Y358N*, *mdtM*, *acrF*	JAOXMQ000000000
DD9	15	AMX, AMP, CFM, CPZ, CTX, CTR, CEP, CIP, E, GEN, LE, MO, PF, SZ, TE	21	*aac(3)-IIe*, *aadA5*, *aac(6′)-Ib-cr5*, *blaEC*, *blaOXA-1*, *blaCTX-M-15*, *tet(A)*, *dfrA17*, *parE_I529L*, *parC_E84V*, *parC_S80I*, *gyrA_D87N*, *gyrA_S83L*, *ptsI_V25I*, *uhpT_E350Q*, *glpT_E448K*, *catB3*, *pmrB_E123D*, *mdtM*, *acrF*, *emrD*	JAOXMR000000000
PH1	9	C, CIP, E, GEN, LE, MO, PF, SPT, TE	19	*aac(3)-IId*, *aph(4)-Ia*, *aac(3)-Iva*, *aadA1*, *aadA2*, *blaEC*, *sul3*, *tet(A)*, *parC_E84G*, *parC_S80I*, *parE_I355T*, *gyrA_D87N*, *gyrA_S83L*, *glpT_E448K*, *uhpT_E350Q*, *cyaA_S352T*, *cmlA1*, *emrD*, *mdtM*	JAOXMT000000000
PD14	11	AMX, AMP, CIP, COT, E, LE, MO, PF, S, SZ, TE	16	*aadA5*, *aph(6)-Id*, *aph(3”)-Ib*, *blaEC*, *blaTEM-1*, *sul2*, *tet(B)*, *dfrA17*, *parC_S80I*, *gyrA_D87N_gyrA_S83L*, *glpT_E448K*, *cyaA_S352T*, *mdtM*, *acrF*, *emrD*	JAOXMS000000000

AMP—Ampicillin; CTX—Cefotaxime; SPT—Spectinomycin; TE—Tetracycline; CPZ-Cefoperazone; GEN-Gentamicin; C—Chloramphenicol; PF—Pefloxacin; CIP—Ciprofloxacin; S—Streptomycin; CFM—Cefixime; AMX—Amoxicillin; CEP—Cephalothin; E—Erythromycin; CTR—Ceftriaxone; COT—Co-trimoxazole; LE—Levofloxacin; MO—Moxifloxacin; and SZ—Sulfadiazine.

**Table 5:** ARGs detected from various antibiotic classes using AMRFinderPlus tool.

Sr. No.	Antibiotic Class	No. of ARGs	Name of Genes
1	AMINOGLYCOSIDE	12	*aph(3″)-Ib*, *aph(6)-Id*, *aadA1*, *ant(2″)-Ia*, *aadA5*, *aph(4)-Ia*, *aadA2*, *aac(3)-Iva*, *aac(3)-Via*, *aac(3)-IId*, *aac(6′)-Ib-cr5*, *aac(3)-IIe*
2	QUINOLONE	8	*qnrS1*, *gyrA_S83L*, *parC_S80I*, *gyrA_D87N*, *parE_I529L*, *parE_I355T*, *parC_E84G*, *parC_E84V*
3	β-LACTAM	5	*bla*EC, *bla*_OXA-1_, *bla*_TEM_, *bla*_CTX-M-15_, *bla*_TEM-1_
4	EFFLUX	4	*acrF*, *emrD*, *mdtM*, *emrE*
5	SULPHONAMIDE	3	*sul1*, *sul2*, *sul3*
6	TRIMETHOPRIM	3	*dfrA5*, *dfrA17*, *dfrA36*
7	FOSFOMYCIN	3	*glpT_E448K*, *uhpT_E350Q*, *ptsI_V25I*
8	PHENICOL	3	*floR*, *catB3*, *cmlA1*
9	TETRACYCLINE	2	*tet(A)*, *tet(B)*
10	COLISTIN	2	*pmrB_Y358N*, *pmrB_E123D*
11	MACROLIDE	1	*mph(A)*
12	FOSMIDOMYCIN	1	*cyaA_S352T*

**Table 6:** Integrons and related gene cassettes found in *E. coli* isolates.

Sample ID	Integrase Gene		No. of Gene Cassettes Present in *intI1*	Name of Gene Cassettes Present in *IntI1*
	*IntI1*	*IntI2*		
BH8	-	-	-	-
BD11	+	-	3	*dhfrA17*, *aadA5*, *QacEdelta1*
CH13	-	-	-	*-*
CD13	+	-	3	*ant(2″)-Ia*, *aadA1*, *emrE*
DH9	+	-	1	*aac(3)-VI*
DD9	+	-	5	*aac(6)-Ib*, *bla*_OXA-1_, *catB*, *aadA*, *dhfrA17*
PH1	+	-	4	*qacL*, *cmlA1*, *aadA2*, *aadA1*
PD14	+	-	2	*aadA5*, *dhfrA17*

“+”, gene detected (Positive); “-”, gene not detected (Negative).

## Data Availability

The *E. coli* genome data can be found in the NCBI database with the accession number PRJNA891125 (*Escherichia coli* (ID 891125))—BioProject—NCBI (nih.gov) (accessed on 22 October 2022)).

## Supplementary Materials

The following supporting information can be downloaded at https://www.mdpi.com/article/10.3390/genes14061212/s1, Figure S1: Structure of class 1 integron; Figure S2: Environmental resistome and the structure of class 1 integron; Figure S3: Lactose fermenting pink coloured colonies of *E. coli* on MacConkey agar; Figure S4: Greenish metallic sheen producing *E. coli* on Eosin-Methylene Blue agar; Figure S5: Isolated pure cultured colonies of *E. coli* on BHI agar; Figure S6: Petridishes showing antibiotic susceptibility pattern of *E. coli* by disc diffusion method (phenotypic method); Figure S7: Percentage of *E. coli* isolates showing sensitive, intermediate and resistance to drug; Figure S8: Representative Electropherogram of DNA library on bioanalyzer; Table S1: Result of Anti-microbial Susceptibility Test; Table S2: Number and percentage of E. coli isolates sensitive, intermediate, and resistant to antibacterial drugs; Table S3: ARGs detected in various antibiotic classes.

## References

[1] Allocati N., Masulli M., Alexeyev M.F., Di Ilio C. (2013). Escherichia coli in Europe: An overview. Int. J. Environ. Res. Public Health.

[2] Poirel L., Madec J.-Y., Lupo A., Schink A.-K., Kieffer N., Nordmann P., Schwarz S. (2018). Antimicrobial resistance in Escherichia coli. Microbiol. Spectr..

[3] Messele Y.E., Alkhallawi M., Veltman T., Trott D.J., McMeniman J.P., Kidd S.P., Low W.Y., Petrovski K.R. (2022). Phenotypic and Genotypic Analysis of Antimicrobial Resistance in Escherichia coli Recovered from Feedlot Beef Cattle in Australia. Animals.

[4] Furuya E.Y., Lowy F.D. (2006). Antimicrobial-resistant bacteria in the community setting. Nat. Rev. Microbiol..

[5] Rybaříková J., Dolejská M., Materna D., Literák I., Čížek A. (2010). Phenotypic and genotypic characteristics of antimicrobial resistant Escherichia coli isolated from symbovine flies, cattle and sympatric insectivorous house martins from a farm in the Czech Republic (2006–2007). Res. Vet. Sci..

[6] Puvača N., de Llanos Frutos R. (2021). Antimicrobial resistance in escherichia coli strains isolated from humans and Pet animals. Antibiotics.

[7] Massella E., Reid C.J., Cummins M.L., Anantanawat K., Zingali T., Serraino A., Piva S., Giacometti F., Djordjevic S.P. (2020). Snapshot study of whole genome sequences of Escherichia coli from healthy companion animals, livestock, wildlife, humans and food in Italy. Antibiotics.

[8] Anjum M.F. (2015). Screening methods for the detection of antimicrobial resistance genes present in bacterial isolates and the microbiota. Future Microbiol..

[9] Sultan I., Rahman S., Jan A.T., Siddiqui M.T., Mondal A.H., Haq Q.M.R. (2018). Antibiotics, resistome and resistance mechanisms: A bacterial perspective. Front. Microbiol..

[10] Bhardwaj A.K., Vinothkumar K., Rajpara N., Mohanty P., Kutar B. (2014). Therapeutic limitations due to antibiotic drug resistance: Road to alternate therapies. Front. Anti-Infect. Drug Discov..

[11] Ploy M.-C., Lambert T., Couty J.-P., Denis F. (2000). Integrons: An antibiotic resistance gene capture and expression system. Clin. Chem. Lab. Med..

[12] Ghaly T.M., Gillings M.R., Penesyan A., Qi Q., Rajabal V., Tetu S.G. (2021). The natural history of integrons. Microorganisms.

[13] Rowe-Magnus D.A., Mazel D. (2002). The role of integrons in antibiotic resistance gene capture. Int. J. Med. Microbiol..

[14] Deng Y., Bao X., Ji L., Chen L., Liu J., Miao J., Chen D., Bian H., Li Y., Yu G. (2015). Resistance integrons: Class 1, 2 and 3 integrons. Ann. Clin. Microbiol. Antimicrob..

[15] Angers-Loustau A., Petrillo M., Bengtsson-Palme J., Berendonk T., Blais B., Chan K.-G., Coque T.M., Hammer P., Heß S., Kagkli D.M. (2018). The challenges of designing a benchmark strategy for bioinformatics pipelines in the identification of antimicrobial resistance determinants using next generation sequencing technologies. F1000Research.

[16] Feldgarden M., Brover V., Gonzalez-Escalona N., Frye J.G., Haendiges J., Haft D.H., Hoffmann M., Pettengill J.B., Prasad A.B., Tillman G.E. (2021). AMRFinderPlus and the Reference Gene Catalog facilitate examination of the genomic links among antimicrobial resistance, stress response, and virulence. Sci. Rep..

[17] Néron B., Littner E., Haudiquet M., Perrin A., Cury J., Rocha E.P. (2022). IntegronFinder 2.0: Identification and analysis of integrons across bacteria, with a focus on antibiotic resistance in Klebsiella. Microorganisms.

[18] Bakhshi M., Fatahi Bafghi M., Astani A., Ranjbar V., Zandi H., Vakili M. (2017). Antimicrobial resistance pattern of Escherichia coli isolated from chickens with colibacillosis in Yazd, Iran. J. Food Qual. Hazards Control.

[19] Touzain F., Le Devendec L., de Boisséson C., Baron S., Jouy E., Perrin-Guyomard A., Blanchard Y., Kempf I. (2018). Characterization of plasmids harboring bla CTX-M and bla CMY genes in E. coli from French broilers. PLoS ONE.

[20] Elsharawy N.T., Al-Zahrani H., El-Waseif A.A. (2022). Phenotypic and Genotypic Characterization of Antimicrobial Resistance in Escherichia coli Isolates from Chicken Meat. J. Food Nutr. Res..

[21] Awad A.M., El-Shall N.A., Khalil D.S., El-Hack M.E.A., Swelum A.A., Mahmoud A.H., Ebaid H., Komany A., Sammour R.H., Sedeik M.E. (2020). Incidence, pathotyping, and antibiotic susceptibility of avian pathogenic Escherichia coli among diseased broiler chicks. Pathogens.

[22] Meshref A.-M.E., Eldesoukey I.E., Alouffi A.S., Alrashedi S.A., Osman S.A., Ahmed A.M. (2021). Molecular analysis of antimicrobial resistance among Enterobacteriaceae isolated from diarrhoeic calves in Egypt. Animals.

[23] Shecho M., Thomas N., Kemal J., Muktar Y. (2017). Cloacael carriage and multidrug resistance Escherichia coli O157: H7 from poultry farms, eastern Ethiopia. J. Vet. Med..

[24] Ammar A., Abd El-Hamid M.I., Eid S.E., El Oksh A.S. (2015). Insights into antimicrobial resistance and virulence genes of emergent multidrug resistant avian pathogenic Escherichia coli in Egypt: How closely related are they. Rev. Med. Vet..

[25] Awosile B., Reyes-Velez J., Cuesta-Astroz Y., Rodríguez-Lecompte J.C., Saab M.E., Heider L.C., Keefe G., Sánchez J., McClure J.T. (2020). Whole-genome sequence analysis of 4 fecal blaCMY-2-producing Escherichia coli isolates from Holstein dairy calves. J. Dairy Sci..

[26] Taviani E., Muchongo A., Kim S.W., Van Kessel J.A.S., Haley B.J. (2021). Genomic Analysis of Antibiotic-Resistant and-Susceptible Escherichia coli Isolated from Bovine Sources in Maputo, Mozambique. Foodborne Pathog. Dis..

[27] Mattioni Marchetti V., Bitar I., Mercato A., Nucleo E., Marchesini F., Mancinelli M., Prati P., Scarsi G.S., Hrabak J., Pagani L. (2020). Deadly Puppy Infection Caused by an MDR Escherichia coli O39 bla CTX–M–15, bla CMY–2, bla DHA–1, and aac (6)-Ib-cr–Positive in a Breeding Kennel in Central Italy. Front. Microbiol..

[28] Haley B.J., Kim S.W., Salaheen S., Hovingh E., Van Kessel J.A.S. (2022). Virulome and genome analyses identify associations between antimicrobial resistance genes and virulence factors in highly drug-resistant Escherichia coli isolated from veal calves. PLoS ONE.

[29] Guo S., Tay M.Y., Aung K.T., Seow K.L., Ng L.C., Purbojati R.W., Drautz-Moses D.I., Schuster S.C., Schlundt J. (2019). Phenotypic and genotypic characterization of antimicrobial resistant Escherichia coli isolated from ready-to-eat food in Singapore using disk diffusion, broth microdilution and whole genome sequencing methods. Food Control.

[30] Roedel A., Vincze S., Projahn M., Roesler U., Robé C., Hammerl J.A., Noll M., Al Dahouk S., Dieckmann R. (2021). Genetic but no phenotypic associations between biocide tolerance and antibiotic resistance in Escherichia coli from German broiler fattening farms. Microorganisms.

[31] Tyson G.H., McDermott P.F., Li C., Chen Y., Tadesse D.A., Mukherjee S., Bodeis-Jones S., Kabera C., Gaines S.A., Loneragan G.H. (2015). WGS accurately predicts antimicrobial resistance in Escherichia coli. J. Antimicrob. Chemother..

[32] Vounba P., Kane Y., Ndiaye C., Arsenault J., Fairbrother J.M., Bada Alambédji R. (2018). Molecular characterization of Escherichia coli isolated from chickens with colibacillosis in Senegal. Foodborne Pathog. Dis..

[33] Cummins M.L., Reid C.J., Chowdhury P.R., Bushell R.N., Esbert N., Tivendale K.A., Noormohammadi A.H., Islam S., Marenda M.S., Browning G.F. (2019). Whole genome sequence analysis of Australian avian pathogenic Escherichia coli that carry the class 1 integrase gene. Microb. Genom..

[34] Hong J.S., Song W., Park H.-M., Oh J.-Y., Chae J.-C., Jeong S., Jeong S.H. (2020). Molecular characterization of fecal extended-spectrum β-lactamase-and AmpC β-lactamase-producing Escherichia coli from healthy companion animals and cohabiting humans in South Korea. Front. Microbiol..

[35] Athanasakopoulou Z., Reinicke M., Diezel C., Sofia M., Chatzopoulos D.C., Braun S.D., Reissig A., Spyrou V., Monecke S., Ehricht R. (2021). Antimicrobial resistance genes in ESBL-producing Escherichia coli isolates from animals in Greece. Antibiotics.

[36] Changkaew K., Utrarachkij F., Siripanichgon K., Nakajima C., Suthienkul O., Suzuki Y. (2014). Characterization of antibiotic resistance in Escherichia coli isolated from shrimps and their environment. J. Food Prot..

[37] Huang X., Yang X., Shi X., Erickson D.L., Nagaraja T., Meng J. (2021). Whole-genome sequencing analysis of uncommon Shiga toxin-producing Escherichia coli from cattle: Virulence gene profiles, antimicrobial resistance predictions, and identification of novel O-serogroups. Food Microbiol..

[38] Afridi O.K., Ali J., Chang J.H. (2020). Next-generation sequencing based gut resistome profiling of broiler chickens infected with multidrug-resistant Escherichia coli. Animals.

[39] Du X., Shen Z., Wu B., Xia S., Shen J. (2005). Characterization of class 1 integrons-mediated antibiotic resistance among calf pathogenic Escherichia coli. FEMS Microbiol. Lett..

[40] Dessie H.K., Bae D.H., Lee Y.J. (2013). Characterization of integrons and their cassettes in Escherichia coli and Salmonella isolates from poultry in Korea. Poult. Sci..

[41] Adelowo O.O., Fagade O.E., Agersø Y. (2014). Antibiotic resistance and resistance genes in Escherichia coli from poultry farms, southwest Nigeria. J. Infect. Dev. Ctries..

