# Supplementary material for: Profiling of Antimicrobial Resistance Genes and Integron from Escherichia coli Isolates Using Whole Genome Sequencing
Source: Genes (Basel). 2023 Jun 1;14(6):1212. doi: 10.3390/genes14061212 (PMC10298372; doi:10.3390/genes14061212)
Supplement: Supplementary file 1 [file genes-14-01212-s001.zip › genes-2349705-supplementary-Final.pdf]

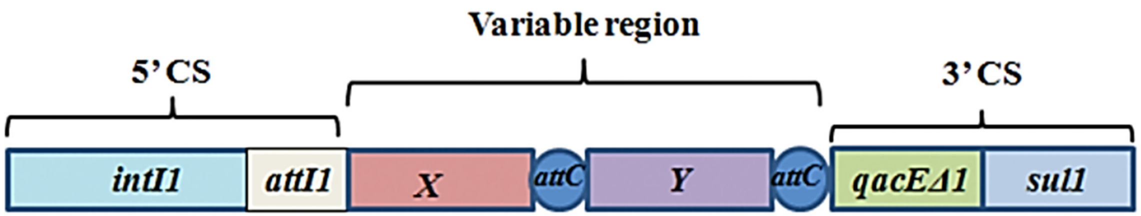

Figure S1. Structure of class 1 integrons.

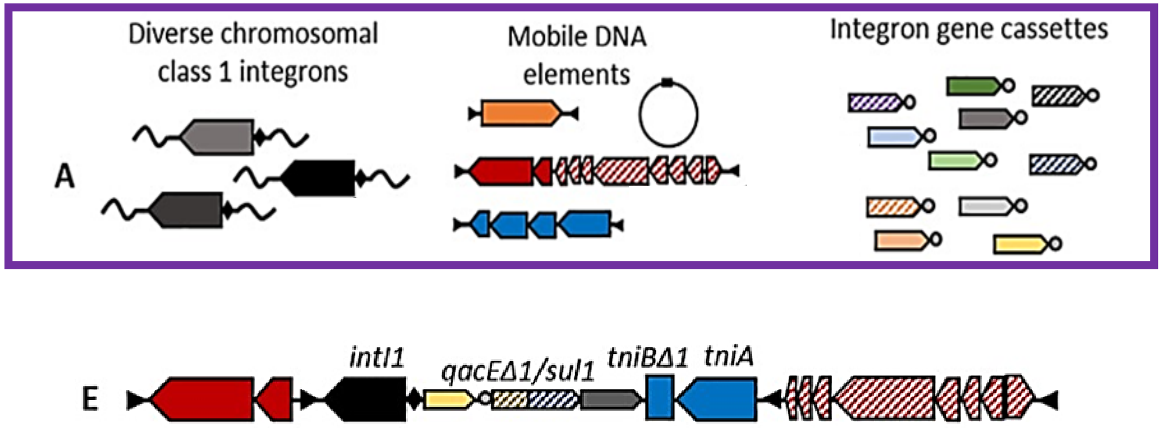

Figure S2. Environmental resistome and the structure of class 1 integron.

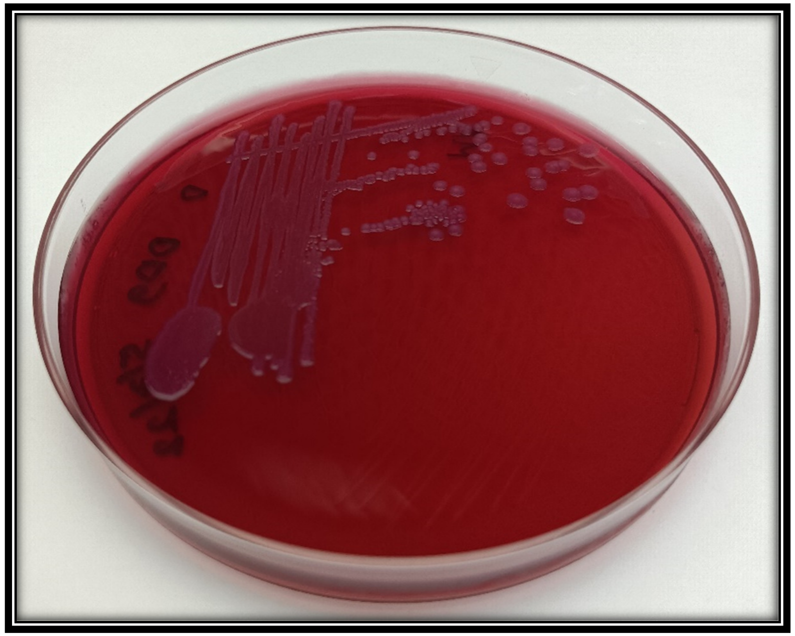

Figure S3. Lactose fermenting pink coloured colonies of *E. coli* on MacConkey agar.

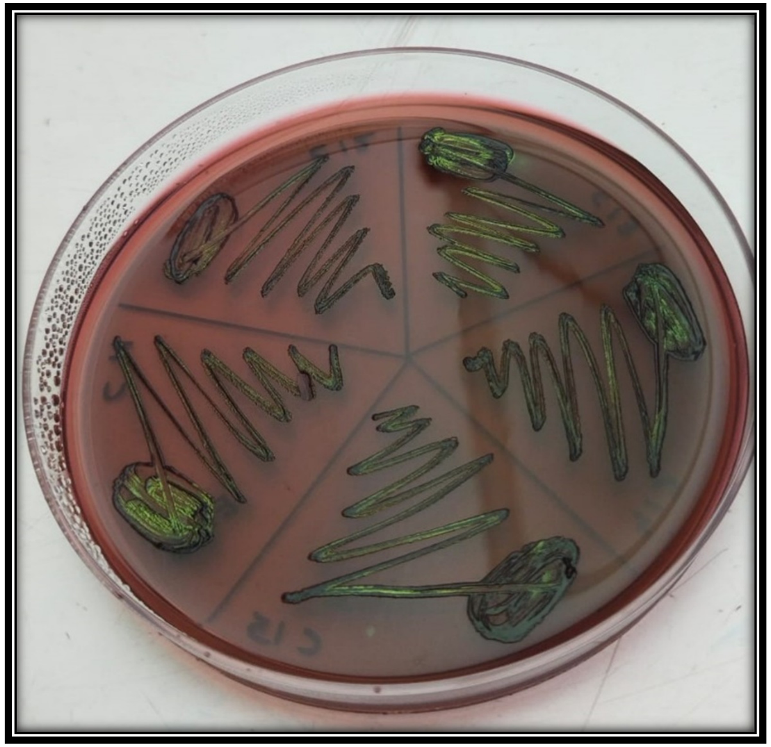

Figure S4. Greenish metallic sheen producing *E. coli* on Eosin-Methylene Blue agar.

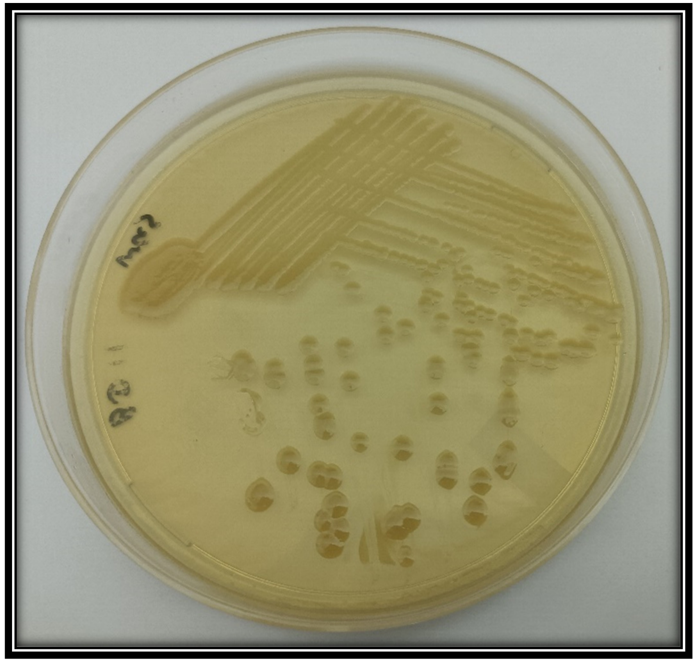

Figure S5. Isolated pure cultured colonies of *E. coli* on BHI agar.

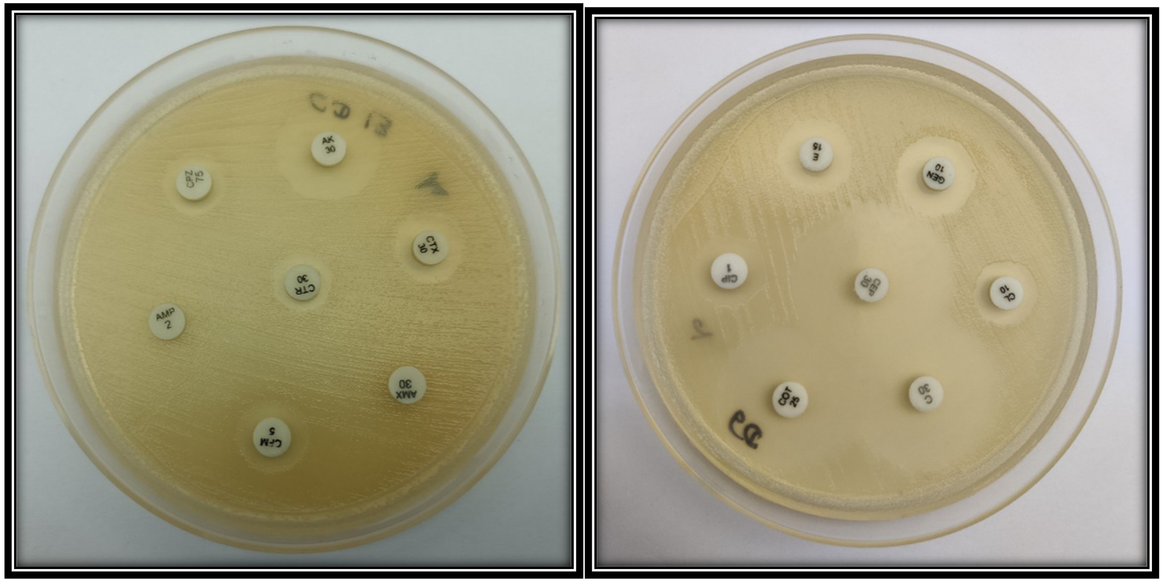

Figure S6. Petridishes showing antibiotic susceptibility pattern of *E. coli* by disc diffusion method (phenotypic method).

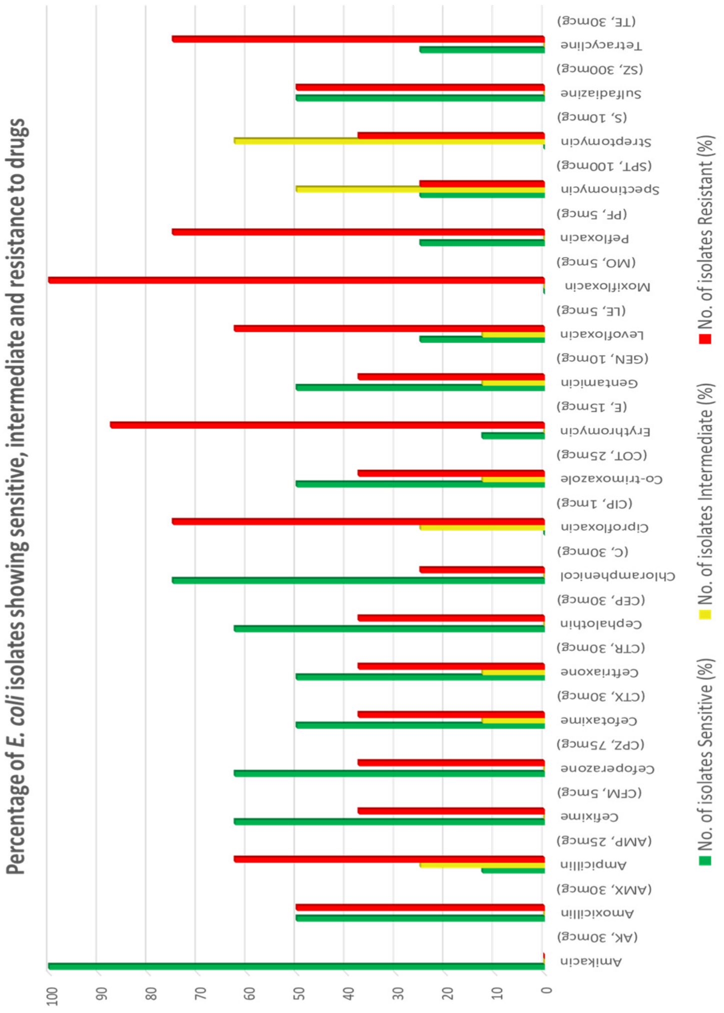

Figure S7. Percentage of *E. coli* isolates showing sensitive, intermediate and resistance to drugs.

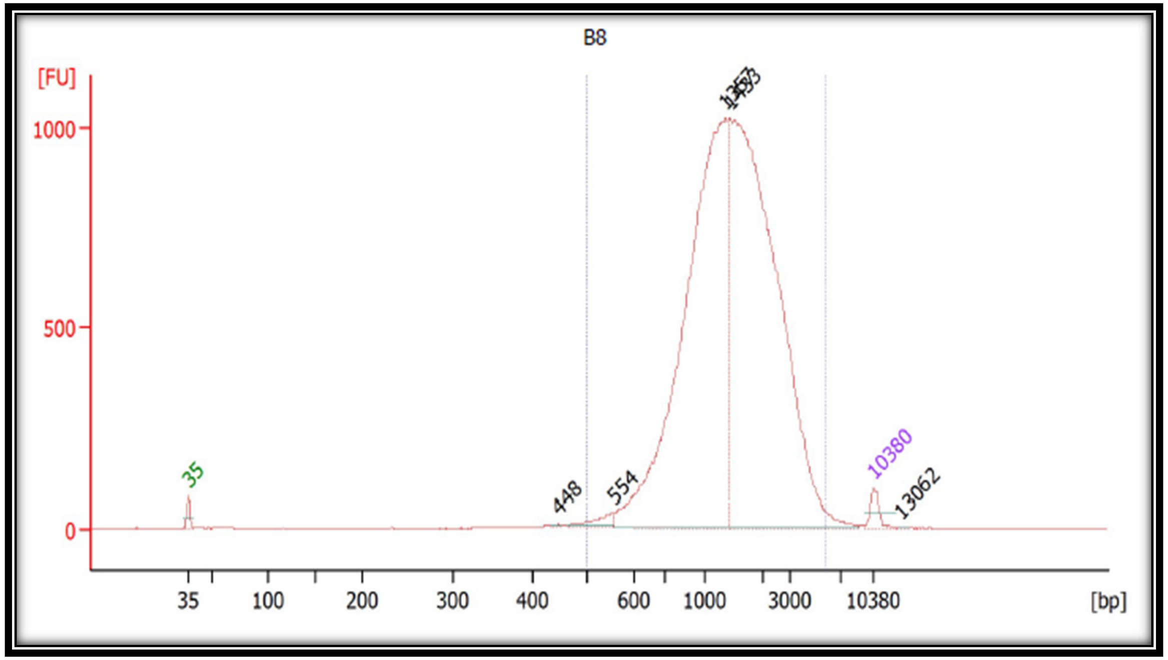

Figure S8. Representative Electropherogram of DNA library on bioanalyzer.

Table S1. Result of Anti-microbial Susceptibility Test.

Name of Antibiotic (Code)	Samples																							
	B8			BD11			C13			CD13			D9			DD9			PH1			P14		
	**S**	**I**	**R**	**S**	**I**	**R**	**S**	**I**	**R**	**S**	**I**	**R**	**S**	**I**	**R**	**S**	**I**	**R**	**S**	**I**	**R**	**S**	**I**	**R**
Amikacin (AK, 30 mcg)	S	-	-	S	-	-	S	-	-	S	-	-	S	-	-	S	-	-	S	-	-	S	-	-
Amoxicillin (AMX, 30mcg)	S	-	-	-	-	R	S	-	-	-	-	R	S	-	-	-	-	R	S	-	-	-	-	R
Ampicillin (AMP, 25 mcg)	-	I	-	-	-	R	-	-	R	-	-	R	S	-	-	-	-	R	-	I	-	-	-	R
Cefixime (CFM, 5mcg)	S	-	-	-	-	R	S	-	-	-	-	R	S	-	-	-	-	R	S	-	-	S	-	-
Cefoparazone (CPZ, 75mcg)	S	-	-	-	-	R	S	-	-	-	-	R	S	-	-	-	-	R	S	-	-	S	-	-
Cefotaxime (CTX, 30mcg)	S	-	-	-	-	R	-	I	-	-	-	R	S	-	-	-	-	R	S	-	-	S	-	-
Ceftriaxone (CTR, 30mcg)		I	-	-	-	R	S	-	-	-	-	R	S	-	-	-	-	R	S	-	-	S	-	-
Cephalothin (CEP, 30mcg)	S	-	-	-	-	R	S	-	-	-	I	-	S	-	-	-	-	R	S	-	-	S	-	-
Chloramphenicol (C, 30mcg)	S	-	-	S	-	-	S	-	-	-	-	R	S	-	-	S	-	-	-	-	R	S	-	-
Ciprofloxacin (CIP, 1mcg)	-	I	-	-	-	R	-	I	-	-	-	R	-	-	R	-	-	R	-	-	R	-	-	R
Colistin (CL, 10 mcg)	-	-	-	-	-	-	-	-	-	-	-	-	-	-	-	-	-	-	-	-	-	-	-	-
Co-trimoxazole (COT, 25 mcg)	S	-	-	-	-	R	S	-	-	-	-	R	S	-	-	-	I	-	S	-	-	-	-	R
Erythromycin (E, 15 mcg)	-	-	R	-	-	R	-	-	R	-	-	R	S	-	-	-	-	R	-	-	R	-	-	R
Gentamicin (GEN, 10mcg)	S	-	-	S	-	-	S	-	-	-	-	R	-	I	-	-	-	R	-	-	R	S	-	-
Levofloxacin (LE, 5mcg)	S	-	-	-	I	-	S	-	-	-	-	R	-	-	R	-	-	R	-	-	R	-	-	R
Moxifloxacin (MO, 5mcg)	-	-	R	-	-	R	-	-	R	-	-	R	-	-	R	-	-	R	-	-	R	-	-	R
Pefloxacin (PF, 5mcg)	S	-	-	-	-	R	S	-	-	-	-	R	-	-	R	-	-	R	-	-	R	-	-	R
Spectinomycin (SPT, 100 mcg)	-	I	-	-	I	-	S	-	-	-	-	R	S	-	-	-	I	-	-	-	R	-	I	-
Streptomycin (S, 10 mcg)	-	I	-	-	-	R	-	I	-	-	-	R	-	I	-	-	I	-	-	I	-	-	-	R
Sulphadiazine (SZ, 300 mcg)	S	-	-	-	-	R	S	-	-	-	-	R	S	-	-	-	-	R	S	-	-	-	-	R

**Tetracycline (TE, 30 mcg)**	S	-	-	-	-	R	S	-	-	-	-	R	-	-	R	-	-	R	-	-	R	-	-	R

Table S-2 . Number and percentage of E. coli isolates sensitive, intermediate, and resistant to antibacterial drugs.

**Name of Antibiotic (Code)**	**No. of isolates**		
	**Sensitive (%)**	**Intermediate (%)**	**Resistant (%)**
Amikacin (AK, 30mcg)	**8/8 (100)**	0/0 (0.00)	0/0 (0.00)
Amoxicillin (AMX, 30mcg)	4/8 (50.00)	0/0 (0.00)	4/8 (50.00)
Ampicillin (AMP, 25mcg)	1/8 (12.50)	2/8 (25.00)	**5/8 (62.50)**
Cefixime (CFM, 5mcg)	**5/8 (62.50)**	0/0 (0.00)	3/8 (37.50)
Cefoperazone (CPZ, 75mcg)	**5/8 (62.50)**	0/0 (0.00)	3/8 (37.50)
Cefotaxime (CTX, 30mcg)	4/8 (50.00)	1/8 (12.50)	3/8 (37.50)
Ceftriaxone (CTR, 30mcg)	4/8 (50.00)	1/8 (12.50)	3/8 (37.50)
Cephalothin (CEP, 30mcg)	**5/8 (62.50)**	0/0 (0.00)	3/8 (37.50)
Chloramphenicol (C, 30mcg)	**6/8 (75.00)**	0/0 (0.00)	2/8 (25.00)
Ciprofloxacin (CIP, 1mcg)	0/0 (0.00)	2/8 (25.00)	**6/8 (75.00)**
Co-trimoxazole (COT, 25mcg)	4/8 (50.00)	1/8 (12.50)	3/8 (37.50)
Erythromycin (E, 15mcg)	1/8 (12.50)	0/0 (0.00)	**7/8 (87.50)**
Gentamicin (GEN, 10mcg)	4/8 (50.00)	1/8 (12.50)	3/8 (37.50)
Levofloxacin (LE, 5mcg)	2/8 (25.00)	1/8 (12.50)	**5/8 (62.50)**
Moxifloxacin (MO, 5mcg)	0/0 (0.00)	0/0 (0.00)	**8/8 (100)**

Pefloxacin (PF, 5mcg)	2/8 (25.00)	0/0 (0.00)	**6/8 (75.00)**
Spectinomycin (SPT, 100mcg)	2/8 (25.00)	4/8 (50.00)	2/8 (25.00)
Streptomycin (S, 10mcg)	0/0 (0.00)	5/8 (62.50)	3/8 (37.50)
Sulfadiazine (SZ, 300mcg)	4/8 (50.00)	0/0 (0.00)	4/8 (50.00)
Tetracycline (TE, 30mcg)	2/8 (25.00)	0/0 (0.00)	**6/8 (75.00)**

Table S3 ARGs detected in various antibiotic classes

Sr No.	Antibiotic class	No. of ARGs	Name of genes	Sample ID	Sample wise No. of ARGs	Sample wise ARGs
1	AMINOGLYCOSIDE	12	*aph(3′′)-Ib, aph(6)-Id, aadA1, ant(2′′)-Ia, aadA5, aph(4)-Ia, aadA2, aac(3)-IVa, aac(3)-VIa, aac(3)-IId, aac(6′)-Ib-cr5, aac(3)-IIe*	B8	0	-
				BD11	3	*aph(3′′)-Ib, aph(6)-Id, aadA5*
				C13	0	-
				CD13	4	*aph(3′′)-Ib, aph(6)-Id, aadA1, ant(2′′)-Ia*
				D9	1	*aac(3)-VIa*
				DD9	3	*aac(3)-IIe, aadA5, aac(6′)-Ib-cr5*
				PH1	5	*aac(3)-IId, aph(4)-Ia, aac(3)-IVa, aadA1, aadA2*
				P14	3	*aadA5, aph(6)-Id, aph(3′′)-Ib*
2	BETA-LACTAM	5	bla_{EC}, $bla_{\text{OXA-1}}$, bla_{TEM}, $bla_{\text{CTX-M-15}}$, $bla_{\text{TEM-1}}$	B8	1	bla_{EC}
				BD11	2	bla_{EC}, $bla_{\text{CTX-M-15}}$
				C13	1	bla_{EC}
				CD13	3	bla_{EC}, bla_{TEM}, $bla_{\text{CTX-M-15}}$
				D9	1	bla_{EC}
				DD9	3	bla_{EC}, $bla_{\text{CTX-M-15}}$, $bla_{\text{OXA-1}}$
				PH1	1	bla_{EC}
				P14	2	bla_{EC}, $bla_{\text{TEM-1}}$

3	SULFONAMIDE	3	*sul1, sul2, sul3*	B8	0	-
				BD11	2	*sul1, sul2*
				C13	0	-
				CD13	2	*sul1, sul2*
				D9	2	*sul1, sul2*
				DD9	0	-
				PH1	1	*sul3*
				P14	1	*sul2*
4	TETRACYCLINE	2	*tet(A), tet(B)*	B8	0	-
				BD11	1	*tet(A)*
				C13	0	-
				CD13	1	*tet(A)*
				D9	1	*tet(A)*
				DD9	1	*tet(A)*
				PH1	1	*tet(A)*
				P14	1	*tet(B)*
5	TRIMETHOPRIM	3	*dfrA5, dfrA17, dfrA36*	B8	0	-
				BD11	1	*dfrA17*
				C13	0	-
				CD13	2	*dfrA5, dfrA36*
				D9	0	-
				DD9	1	*dfrA17*
				PH1	0	-
				P14	1	*dfrA17*
6	QUINOLONE	8		B8	0	-

			qnrS1, gyrA_S83L, parC_S80I, gyrA_D87N, parE_I529L, parE_I355T, parC_E84G, parC_E84V	BD11	1	*qnrS1*
				C13	0	-
				CD13	2	*qnrS1, gyrA_S83L*
				D9	3	*parC_S80I, gyrA_D87N, gyrA_S83L*
				DD9	5	*parE_I529L, parC_E84V, parC_S80I, gyrA_D87N, gyrA_S83L*
				PH1	5	*parC_E84G, parC_S80I, parE_I355T, gyrA_D87N, gyrA_S83L*
				P14	3	parC_S80I, gyrA_D87N, gyrA_S83L
7	FOSFOMYCIN	3	*glpT_E448K, uhpT_E350Q, ptsI_V25I*	B8	1	*glpT_E448K*
				BD11	1	*glpT_E448K*
				C13	1	*glpT_E448K*
				CD13	1	*glpT_E448K*
				D9	2	*glpT_E448K, uhpT_E350Q*
				DD9	3	*glpT_E448K, uhpT_E350Q, ptsI_V25I*
				PH1	2	*glpT_E448K, uhpT_E350Q*
				P14	1	*glpT_E448K*
8	PHENICOL	3	*floR, catB3, cmlA1*	B8	0	-
				BD11	0	-
				C13	0	-
				CD13	1	*floR*

				D9	0	-
				DD9	1	*catB3*
				PH1	1	*cmlA1*
				P14	0	-
9	MACROLIDE	1	*mph(A)*	B8	0	-
				BD11	1	*mph(A)*
				C13	0	-
				CD13	0	-
				D9	0	-
				DD9	0	-
				PH1	0	-
				P14	0	-
10	COLISTIN	2	*pmrB_Y358N,pmrB_E123D*	B8	0	-
				BD11	1	*pmrB_Y358N*
				C13	1	*pmrB_Y358N*
				CD13	0	-
				D9	1	*pmrB_Y358N*
				DD9	1	*pmrB_E123D*
				PH1	0	-
				P14	0	-
11	FOSMIDOMYCIN	1	*cyaA_S352T*	B8	0	-
				BD11	0	-
				C13	0	-
				CD13	0	-
				D9	0	-

				DD9	0	-
				PH1	1	*cyaA_S352T*
				P14	1	*cyaA_S352T*
12	EFFLUX	3	*acrF, emrD, mdtM*	B8	3	*emrD,mdtM, acrF*
				BD11	2	*mdtM, acrF*
				C13	2	*mdtM, acrF*
				CD13	2	*mdtM, acrF*
				D9	2	*mdtM, acrF*
				DD9	3	*emrD,mdtM, acrF*
				PH1	2	*emrD,mdtM*
				P14	3	*emrD,mdtM, acrF*
